# The effect of adipose-derived mesenchymal stem cells on renal function and histopathology in a rat model of ischemia-reperfusion induced acute kidney injury

**DOI:** 10.22038/ijbms.2020.40334.9601

**Published:** 2020-08

**Authors:** Saeed Changizi-Ashtiyani, Leila Hafazeh, Faezeh Ghasemi, Houshang Najafi, Saeed Babaei, Farideh JalallyMashayekhi, Seyed Javad Hoseini, Bahar Bastani

**Affiliations:** 1 Department of Physiology, Arak University of Medical Sciences, Arak, Iran; 2 Blood Transfusion Research Center, High Institute for Research and Education in Transfusion Medicine, Tehran, Iran; 3 Medical Biology Research Center, Kermanshah University of Medical Sciences, Kermanshah, Iran; 4 Department of Anatomy, School of Medicine, Arak University of Medical Sciences, Arak, Iran; 5 Department of Genetics and Biochemistry, School of Medicine, Arak University of MedicalSciences, Arak, Iran; 6 Department of Medical Biochemistry, School of Medicine, Mashhad University of Medical Sciences, Mashhad, Iran; 7 Division of Nephrology, Department of Medicine, Saint Louis University, School of Medicine, Saint Louis, Missouri, USA

**Keywords:** Acute kidney injury, Adipose tissue, Ischemia-reperfusion injury, Mesenchymal stem cells, Rats

## Abstract

**Objective(s):**

It has been shown that adipose-derived mesenchymal stem cells (AD-MSC) have protective effects in acute kidney injury (AKI). This study was conducted to assess the therapeutic effects of AD-MSC in rats subjected to acute kidney injury by 45 min of renal ischemia followed by 48 hr of reperfusion (I/R).

**Materials and Methods:**

28 male Wistar rats were divided into four groups, including control, 48-hr sham, 48-hr I/R, and 48-hr I/R receiving AD-MSC. After 48 hr of reperfusion, blood samples were taken from rats’ hearts, and 24-hr urines were collected using a metabolic cage. Serum creatinine level (Cr), blood urea nitrogen (BUN), creatinine clearance (Ccr), absolute sodium excretion (UNaV°), fractional sodium excretion (FENa), absolute potassium excretion (UKV°), factional potassium excretion (FEK), and urine osmolarity were measured. Malondialdehyde (MDA) and ferric reducing antioxidant power (FRAP) levels were measured in the right kidney, while the left kidney was used for histologic study after Hematoxylin-Eosin staining.

**Results:**

Renal I/R significantly increased serum Cr, BUN, UNaV°, FENa, FEK, and tissue MDA, and significantly decreased Ccr and urine osmolarity as compared with the sham group. Moreover, histologic studies showed that I/R increased Bowman capsule area, tubular necrosis, vascular congestion, and caused formation of intratubular casts. Administration of AD-MSC at the time of I/R completely or partially protected kidneys from these I/R induced injuries.

**Conclusion:**

Our results show that injection of AD-MSC can reduce degree of renal injury caused by 45 min of ischemia followed by 48 hr of reperfusion in rats.

## Introduction

The study of kidney diseases has a long history in medicine, including the Persian medicine (1). Despite advanced studies in the recent decades, as yet no definite treatment is available for acute kidney injury (2). Energy crisis in kidney due to ischemia in combination with vascular spasm, endothelial damage, and activation of inflammatory processes lead to rapid cellular death through necrosis and apoptosis (3, 4). Studies performed on rats showed that vascular function remains abnormal up to several days after I/R, which is associated with diminished endothelial nitric oxide production and increased vascular permeability causing platelet and leukocyte aggregation in vascular endothelium, all of which would further aggravate the situation (5).

According to the pathophysiology of AKI, different therapies have been proposed with many limitations. Currently, kidney transplantation and dialysis are used at the end stages of kidney failure, which are associated with their own limitations, such as, excessive cost, requiring surgical procedures, chance of infection, rapid progression of cardiovascular disease, and severe organ shortage (6, 7). A novel treatment could be promoting tissue regeneration with stem cell transplant. Stem cells are undifferentiated cells with the ability of self-renewal and differentiation to different mature cells such as osteocytes, chondrocytes, adipocytes, and myelocytes (8). Using mesenchymal stems cells (MSCs) for treatment of AKI has recently attracted much attention (9). Several studies in the recent years have evaluated the effectiveness of using stem cells in the treatment of AKI in rats. A study showed that intravenous injection of AD-MSC had anti-oxidant and anti-apoptotic effects against renal injuries caused by 45 min of ischemia and 48 hr of reperfusion in rat kidney (10). Similarly, it was reported that intra-arterial injection of AD-MSC reduced inflammation and tissue damage in kidney after 40 min of ischemia and 48 hr of reperfusion (11). Although most studies have suggested positive therapeutic effect of MSCs on AKI, there is lack of detailed information on the optimal method and duration of treatment, number of injected cells, the best time of injection after injury, and its effect on renal function tests and the degree of tissue repair (12). The present study was conducted to evaluate the effectiveness of MSCs transplantation in protecting renal function and histopathology, and oxidative stress at 48 hr after kidney ischemic injury.

## Materials and Methods

***Animals***

This experimental study was conducted on 28 male Wistar rats, weighting 180–200 g. Adult rats were obtained from the Animal Laboratory Facility, School of Medicine, Arak University of Medical Sciences, and were considered healthy donors for AD-MSCs. We performed all of the experiments in accordance with the approved codes of working with laboratory animals by Ministry of Health and Medical Education (IR.ARAKMU.REC.1396.13). The experimental rats were kept at room temperature (22–24 ^°^C) with 12 hr of light/darkness and had free access to water and food.

***Isolation and determination of AD-MSCs***

Rats were anesthetized by intraperitoneal injection of 60 mg/kg ketamine hydrochloride (Trittau, Germany) and 6 mg/kg xylazine hydrochloride (Woerden, Netherlands). Rat AD-MSC were isolated and characterized, as described previously (13).

***Study protocol and experimental groups***

Animals were randomly divided into 4 groups (n=7 in each group). The control group did not receive any intervention except for a single infusion of 600 μl PBS into their tail veins. The sham 48-hr group underwent laparotomy but ischemia was not induced and after the operation 1 ml PBS was injected into their tail vein. In the ischemia-reperfusion (I/R-48 hr+PBS) group, vessels of both kidneys were completely clamped for 45 min (14) and 1 ml PBS was injected into their tail vein just after ischemia, followed by 48 hr of blood reperfusion. The fourth group, ischemia-reperfusion preconditioned with MSCs (I/R-48 hr+MSC), received 2×10^6 ^AD-MSCs in 1 ml PBS injected very slowly into their tail vein, followed by 45 min of ischemia and 48 hr of reperfusion. 

***Method of inducing acute kidney injury***

Rats were anesthetized by intraperitoneal injection of 50 mg/kg.bw sodium pentobarbital (Lund beck, Denmark). Rat abdomens were opened with a longitudinal incision in a sterile fashion. Right and left kidney vessels were clamped for 45 min to induce complete ischemia (15). After the operation and unclamping of the vessels, rats were sent back to their cages for 48 hr of recovery during which they had free access to water and standard food.

***Sample collection method***

In the last 24 hr of reperfusion recovery, rats were sent to metabolic cages and their urine was measured by gravimetry, and their urine volume was measured (16). After 48 hr of reperfusion recovery, rats were anesthetized again, blood samples were collected from their hearts and serum was separated; then kidneys were extracted. The left kidneys were decapsulated and put in 10% formalin solution for Hematoxylin-Eosin (H&E) staining and histologic studies. The right kidneys were snap frozen for later assessment of oxidative stress through measuring malondialdehyde (MDA) and ferric reducing anti-oxidant power (FRAP) levels.

***Biochemical analysis***

Blood urea nitrogen (BUN) and serum creatinine (Cr) were measured using an autoanalyzer (Biotechnical, Italy). Urine sodium and potassium were measured using a flame photometer (BWBTech, England). Urine osmolarity was measured by using an osmometer (Osmomat030, Germany). Creatinine clearance (Ccr), absolute and fractional excretion of sodium (U_Na_V^°^ and FE_Na_), and potassium (U_K_V^°^ and FE_K_) were calculated (17).

***Measuring oxidative stress***

To assess oxidative stress status, level of membrane lipid peroxidation was measured with thiobarbituric acid (TBA) method. Kidney samples were homogenized in cold PBS, 200 μl of the homogenized solution was added to a tube containing 20% acetic acid, 0.8% thiobarbituric acid, and 8.1% sodium dodecyl sulfate (SDS) and heated in a water bath (DUBNOFF, USA) at 95 ^°^C for 60 min. After cooling and adding 4 ml of n-butanol, it was centrifuged (4000 rpm) and light absorbance of the upper layer was measured at 532 nm of wavelength using a spectrophotometer (Spectrolab 7500 UV, England). Tetraethoxypropane (TEP) was used as external standard (18).

Anti-oxidant capacity was measured by assessing FRAP. For this purpose, a solution was initially prepared using a mixture of 300 mmol/l acetate buffer (pH=3.6), 10 mM of TPTZ solution (Merck, Germany) in chloride acid (40 mmol/l), and ferric chloride solution (20 mmol/l). Then, 1.5 ml of the above solution was raised to a temperature of 37 ^°^C. The amount of 50 μl of tissue supernatant was added to the above solution to initiate the reaction. Absorption changes were measured at 593 nm. To draw the standard curve, ferrous sulfate. 7H_2_O_2_ (FeSO_4_·7H_2_O_2_) was used, FRAP values were reported as µmol/l (19).

***Histologic studies***

To evaluate the degree of damage to kidney tissue, cortex, outer medulla, and inner medulla were studied using light microscopy. Tissue damage grading was performed in terms of Bowman space enlargement, cell necrosis, vascular congestion, and cast formation in tubules. The highest degree of Bowman space enlargement was compared with the sham group and was graded as 100% damage; the other rats were compared accordingly. Cell necrosis, vascular congestion, and tubular cast formation were graded according to the percentage of involved area (20).

***Statistical analysis***

SPSS version 18 was used for data analysis. Data are reported as mean±SE. *P*<0.05 was considered significant. One-way analysis of variance (ANOVA) and Duncan *post hoc* test were used for data analysis. The precise *P*-value was calculated using the LSD test. Data regarding tissue damage was analyzed using Kruskal Wallis and Man Whitney non-parametric tests.

## Results

***Changes in hemodynamics and renal function***

Ischemia reperfusion (I/R) significantly decreased renal function as evidenced by a significant rise in BUN and serum Cr, and a significant decrease in urine osmolarity (*P*<0.001) and Ccr (*P*<0.01) as compared with the sham group. Additionally, U_Na_V^°^, FE_Na_, and FE_K _were significantly higher in the I/R group as compared with the sham group (Table 1).

Pretreatment with AD-MSC significantly improved Ccr and urine osmolarity as compared with I/R group (IR+stem vs IR+PBS, *P*<0.01). Moreover, FE_Na_, FE_K_, serum Cr, and BUN were all significantly reduced in the AD-MSC treated I/R group as compared with the I/R+PBS group (*P*<0.001) (Table1).

***Changes in oxidative stress and anti-oxidant state of the kidney***

**Figure 1:**
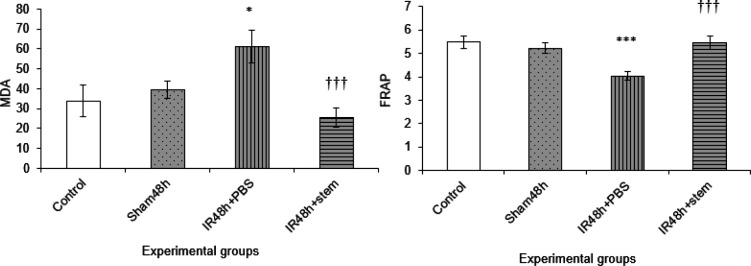
Comparison of mean malondialdehyde and the ferric reducing anti-oxidant power levels in different groups following 45 min renal ischemia and 48 hr reperfusion in rats

In oxidative stress studies, we found a significant increase in MDA level in the I/R group, as compared with the sham group, after 48 hr of reperfusion (*P*<0.05). We also observed that AD-MSC infusion ameliorated any rise in MDA level after I/R (IR48 hr+stem vs IR+PBS, *P*<0.001) (Figure 1).

In the anti-oxidant studies, we found a significant reduction of FRAP in the I/R group as compared with sham group (*P*<0.001). Treatment with AD-MSC significantly increased FRAP levels to the sham group (IR48hr+stem vs IR+PBS, *P*<0.001) (Figure 1).

***Histological changes***

**Figure 2:**
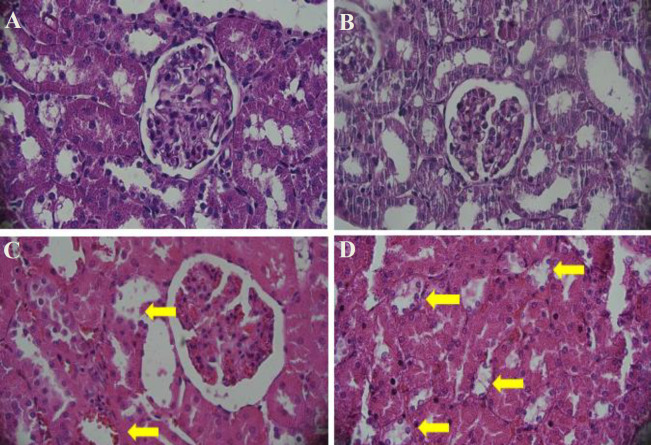
Kidney cross-sections to show Bowman space, vascular congestion, and cellular necrosis in different experimental groups. a) rats with no intervention (control); b) rats undergone surgery without renal vessel clamping and receiving PBS solution and equivalent 48-hr reperfusion period (Sham); c) rats with 45 min of ischemia and 48 hr of reperfusion receiving PBS (I/R 48 hr + PBS), yellow arrows show vascular congestion; and d) rats with 45 min of ischemia and 48 hr of reperfusion pretreated with 2 × 10^6^ AD-MSC (I/R 48 hr + stem cell), yellow arrows show damaged thick ascending limbs of Henle’s loops. 400x magnification; Hematoxylin-Eosin staining

IR: ischemia-reperfusion; AD-MSC: adipose-derived mesenchymal stem cells

I/R resulted in significant total tissue damage as compared with the sham group (*P*<0.001). Forty-five minutes of ischemia followed by 48 hr of reperfusion (I/R-48 hr group) caused significant enlargement of Bowman space, grade 3.35, as compared with the sham group. In the I/R-48 hr group, proximal tubule damage grade in the cortex was 1.61 and cortical thick ascending limb of loop of Henle damage grade was 1.23; in the outer medulla, the degree of damage to pars recta, medullary thick ascending limb of loop of Henle, vascular congestion, and tubular cast formation were 2.37, 2.67, 1.54, and 1.14, respectively; in the inner medulla, the degrees of vascular congestion and tubular cast formation were 1.02 and 1.13, respectively. Pre-treatment with AD-MSC significantly reduced the degree of tissue damage in the outer and inner medulla, but not in the cortex. Total histopathologic damage grade of 16.06 in I/R-48 hr group was significantly reduced to 13.05 after treatment with AD-MSC (*P*<0.01), (Figure 2, Table 2).

## Discussion

Ischemia and reperfusion is a major cause of AKI. There has been a significant interest in using MSCs to treat AKI in recent years. In the present study we sought to investigate the effect of adipose-derived mesenchymal stem cells (AD-MSCs) on an I/R model of AKI, since extracting AD-MSCs is more feasible and less invasive, provides more cells, and can be used more easily in clinical practice. Our results show significant improvement in renal function parameters, lesser oxidative stress, and prevention of kidney tissue damage at 48 hr after AD-MSC injection.

In the present study, I/R caused significant kidney injury at 48 hr after reperfusion, as evidenced by a significant decline in Ccr and significant increase in serum creatinine and BUN. Depletion of tissue ATP storage during ischemia disrupts tight junctions and adherence junctions, which would lead to loss of cell polarity, increased tissue permeability, transposition of Na^+^/K^+^-ATPase from basolateral membrane to apical membrane that would disrupt transcellular sodium transport and increase sodium delivery to distal tubules (21). Increased sodium delivery to macula densa causes vasoconstriction of the afferent arterioles, via adenosine A1 receptors, and that leads to lowering of GFR. Moreover, vasoconstriction of the afferent arterioles decreases glomerular plasma flow rate and glomerular pressure, which in combination with a concomitant ischemia induced mesangial cell contraction decrease filtration coefficient (*k*_f_) (22). In the present study we showed that intravenous injection of AD-MSCs improved several renal function and histopathologic parameters probably by reducing congestion and anti-oxidant and anti-inflammatory effects (23). A study reported the effectiveness of bone marrow MSC injection in improving structure and function of kidney after 40 min of ischemia by lowering inflammation and apoptosis, as well as, by increasing mesenchymal cell proliferation (24) Any treatment that would improve recovery of hemodynamic disturbances caused by I/R could potentially prevent depletion of ATP resources and minimize renal injury. Beiral *et al.* have shown that bone marrow derived stem cells can almost completely preserve mitochondrial ATP synthesis and reduce mitochondrial ROS formation in rat kidneys that were subjected to I/R (25). Moreover, it is shown that MSC derived exosomes contain the key enzymes in the glycolysis step in ATP synthesis (26). Also, the effectiveness of MSCs on preserving tight junctions has been shown before. It was shown that in an ischemic rat bowel model MSCs improve tight junctions, probably by preventing release of TNF-α (27). The observed protective effects of AD-MSC on kidney tight junctions may be explained by the same mechanism.

In the present study, in the I/R 48 hr group urine osmolarity was reduced due to inability of kidneys to concentrate urine and presumably a lower concentration of osmotic particles in kidney medulla (28), while FE_Na_, FE_K_, and U_Na_V^°^ increased. Forty-eight hours after I/R in the group of rats pretreated with AD-MSC urine osmolarity had increased, and FE_Na_ and FE_K _had decreased as compared with the I/R 48hr group. Cells in the pars recta of proximal tubules and in the thick ascending limb of the loop of Henle were significantly damaged by ischemia, which in turn would increase fractional excretion rate of sodium and potassium (29). Seguro *et al.* have shown that reduced activity of Na^+^/K^+^-ATPase in the inner medulla reduces activity of NaCl transporter and is responsible for the observed increase in sodium excretion after ischemia (30).

The observed preferential damage to the thick ascending limb of Henle’s loop after a brief ischemia is due to insufficient oxygen supply by the medullary vasculature and a very high basal metabolic rate and oxygen demand in this nephron segment that is charged with active NaCl transport. It has been shown that ischemia decreases Na^+^/K^+^-ATPase of the inner stripe of the outer medulla, where the medullary thick ascending limbs of Henle’s loop are located, and leads to increased natriuresis, kaliuresis, and polyuria, and that potassium depletion aggravates these derangements (30). Injection of fetal membranes-derived human MSCs in rats who had undergone 45 min of renal ischemia improved functional and morphological recovery through paracrine mechanisms, secretion of various cytokines, and by reducing inflammation (31). Alterations in the activity of renal tubular potassium channels have been shown to be involved in worsening of cellular damage during ischemia. Cytokines, such as IFN-γ and IL-1β may affect the activity of potassium channels in immune cells and renal tubular cells (32). Inhibitory effect of MSC on expression of IFN-γ and IL-1β inflammatory cytokines has been reported (33). Thus, it is possible that the reduced potassium excretion in AD-MSC group is due to the anti-inflammatory effects of AD-MSCs (23). Another mechanism involved in renal function disturbance is activation of NR1 subunit of NMDA (N-methyl-D-aspartate) receptor. Activation of NR1 during ischemia reperfusion reduced GFR, decreased urine volume, and increased sodium and potassium excretion during ischemia and reperfusion (34).

A prolonged period of ATP depletion would lead to biochemical changes, cellular necrosis, and generation of reactive oxygen species (ROS). During reperfusion, the abrupt increase in oxygen supply initiates oxidative reactions (33). Oxidative stress and increased ROS are involved in the pathophysiology of I/R induced renal injury (35, 36). In the present study, MDA level increased in I/R 24 hr group as compared with the sham group, and injection of AD-MSC could ameliorate the rise in MDA after I/R. Recent studies have shown anti-oxidant effects of MSC. Researchers indicated that AD-MSC can reduce renal damage after I/R through increasing expression of heme oxygenase-1 (HO-1) and NAD(P)H quinone oxidoreductase-1 (23). Moreover, it was suggested that MSCs can indirectly decrease ROS, which would lower the level of MDA (37). Since MSCs can reduce the BUN level and elevated BUN can increase ROS and oxidative stress, (38) AD-MSC can decrease MDA through the aforementioned mechanisms. Additionally, expression of Nrf2 (nuclear factor erythroid-2 related factor 2) by MSCs protected them from cell death and apoptosis triggered by hypoxia and oxidative stress conditions (39). Also, proteomic analysis and ELISA have shown that AD-MSC have anti-oxidant activity by expression of proteins such as super oxide dismutase (SOD), insulin like growth factor (IGF), fibroblast growth factor (FGF), pigment epithelium-derived factor (PEDF), hepatocyte growth factor (HGF), and ILs, all of which have anti-oxidant effects (40).

In our study I/R resulted in a significant reduction in FRAP, as compared with the sham group, and intravenous injection of AD-MSC prevented any decline in FRAP after I/R48 hr, confirming the anti-oxidant effect of AD-MSC. Thus, it appears that AD-MSC with their anti-oxidant properties could reduce MDA and increase FRAP by inhibiting the production of reactive oxygen species. Moreover, I/R increased size of the Bowman capsule in the cortex, damaged proximal tubules and TALs in the cortex and outer medulla, and caused vascular congestion and proteinaceous casts that resulted in a total histopathologic damage degree that was significantly higher in the I/R group as compared with the sham group. These histopathologic damages may be caused by release of inflammatory cytokines, over expression of adhesion molecules, and tissue leukocyte infiltration (41). Recent studies have shown that injection of stem cells is effective in treatment of AKI and I/R induced kidney damage. Complex therapeutic mechanisms including anti-inflammatory reactions, anti-oxidant activities, angiogenesis, attenuation of immune response and immobilization of stems cells have been implicated in this process (42, 43). In the present studies reduction of tissue damage in cortex and medulla was observed in the I/R + AD-MSC as compared with I/R + PBS group. In a report, after injection of MSCs into ischemic kidney, the infused MSCs were reported to reside predominantly in the cortex, in glomerular capillaries, and not in the tubules and interstitium or vascular endothelial cells (44). Thus, direct effect of MSCs in tissue repair is unlikely. MSCs may exert their effects by secreting growth factors including IGF-1, HGF, VEGF, and substances with paracrine effects (45). Zhuo *et al.* reported that injection of BM-MSC after 60 min of vascular clamp improved kidney damage by reducing inflammatory cytokines such as TNF-α and IL-6 and increasing VEGF level (37). Endothelial cells play an important role in maintaining vascular tone, leukocyte function, and smooth muscle response to vasoactive agents. Ischemia damages endothelial cells, and thus, the vasodilation response of arterioles to acetylcholine and nitric oxide, and bradykinin diminishes after ischemic injury. Increased vasoconstriction results in further local ischemia in medulla, which activates leukocyte-endothelial interaction and tissue edema, and subsequent damage to pars recta and thick ascending limb of loop of Henle (46). Leukocyte-endothelial interaction is due to presence of adherence molecules such as ICAM-1 on endothelium, which activates leukocytes in inflammatory condition (47). It is shown that bone marrow-derived MSCs decrease ICAM-1, which can subsequently prevent leukocyte infiltration (48). Moreover, physiologic concentrations of NO inhibit expression of ICAM-1 and aggregation of macrophage/monocyte, while higher concentrations of NO can worsen kidney injury (49). NO production increases in ischemia and the produced NO can transform into superoxide to form peroxynitrite anion, which can cause cell death by oxidant damage (49). It has been shown that iNOS is responsible for increased NO level during ischemia, (50) and a study showed that MSCs can suppress NO production, (51) thus, inhibition of NO production by iNOS after injection of MSCs may play a vital role in prevention of cell death due to I/R. Recently it is suggested that secretion of MSC-derived extracellular vesicles (EVS) has a significant role in therapeutic effectiveness of MSCs. Microvesicles may have a protective effect on kidneys by attenuating immune response and anti-apoptotic properties (52). Despite several studies investigating the efficacy of mesenchymal stem cells in ameliorating I/R induced injury in the kidney (53, 54), there are still many uncertainties regarding the number of cells used, type of AKI, the location and time of injection, and the rate of recovery (55, 23). Therefore, the present study was unique in terms of the number of mesenchymal stem cells used, the pattern of renal injury (I/R and 48 hr reperfusion) and the severity of injury, as well as, the time of evaluation after ischemia.

**Table 1:** Renal functional changes 48 hr after ischemia-reperfusion in rats, and the therapeutic effect of adipose-derived mesenchymal stem cells on them

Parameter	Control	Sham 48hr	I/R48hr+PBS	I/R48hr+stem cell
Plasma creatinine concentration (mg/dl)	0.52 ± 0.014	0.57 ±0.008	2.37 ± 0.13 ***	0.94 ± 0.07 ***†††
Blood urea nitrogen (mg/dl)	23.6 ± 1.65	24.12 ± 0.83	84 ± 6.29 ***	34.12 ± 2.66 †††
Absolute urinary sodium excretion (mmol/min/kg)	2.56 ± 0.49	1.81 ± 0.36	3.73 ± 0.68 **	3.87 ± 0.57 **
Absolute urinary potassium excretion (mmol/min/kg)	4.01 ± 0.96	2.82 ± 0.59	2.9 ± 0.63	3.48 ± 0.68
Fraction urinary sodium exertion (mmol/min/kg)	1.33 ± 0.3	0.72 ± 0.07	7.07 ± 1.35 ***	1.46 ± 0.14 †††
Fraction urinary potassium exertion (mmol/min/kg)	72.19 ± 25.74	33.6 ± 1.75	130.42 ± 18.72 **	43.5 ± 5.22 ††
Urine osmolarity (mOsm/kg H_2_O)	1751.7 ± 119.64	1969.7 ± 144.2	744.8 ± 89.13 ***	1231.11 ± 105.11 *** ††
Creatinine clearance (ml/min/kg)	1.67 ± 0.38	1.66 ±0.3	0.54 ± 0.17 **	1.86 ± 0.21††

**P<0.01, ***P<0.001 in comparison with the sham group

††*P*<0.01, †††*P*<0.001 in comparison with the I/R group

IR: ischemia-reperfusion

*P<0.05, ***P<0.001 in comparison with the sham group

†††*P*<0.001 in comparison with the I/R group

IR: ischemia-reperfusion

**Table 2:** Summary of the degree of kidney histopathologic damages induced by 45 min of ischemia followed by 48 hr of reperfusion, and the therapeutic effect of adipose-derived mesenchymal stem cells on them

Histopathologic damages	Study groups			
	Control	Sham 48 hr	I/R 48hr +PBS	I/R 48hr + stem cell
Bowman capsule	0	0.01	3.35	3.65
proximal tubal	0	0	1.61	1.89
thick ascending limb of Henle’s loop	0	0	1.23	1.11
Pars Recta	0	0.01	2.37	1.76
thick ascending limb of Henle’s loop	0	0.02	2.67	2.36
vascular congestion	0	0	1.54	0.78
tubular protein cast	0	0	1.14	0.53
vascular congestion	0	0	1.02	0.51
tubular protein cast	0	0	1.13	0.46
Total histopathologic scale	0	0.04	16.06*	13.05*†

Grades of histopathologic injuries in rats with no intervention (control); rats that underwent laparotomy without clamping of renal vessels and received PBS solution and equivalent 48-hr perfusion period (Sham); rats with 45 min of ischemia and 48 hr of reperfusion that received PBS (I/R 48 hr+ PBS); rats with 45 min of ischemia and 48 hr of reperfusion that had received 2×10^6^ adipose-derived mesenchymal stem cells (I/R 48 hr+stem cell)

*P<0.001 in comparison with the sham group

†*P*<0.01 in comparison with the I/R group

IR: ischemia-reperfusion

## Conclusion

The present experiments show that 45 min of ischemia followed by 48 hr of reperfusion resulted in significant disturbance in kidney function, tissue and cellular damage, and that injection of 2 × 10^6^ AD-MSC into the tail vein can significantly ameliorate these pathophysiologic changes. The protective effect of AD-MSC is probably exerted via paracrine factors, anti-oxidant effects, release of growth factors and anti-inflammatory effects, although, further studies are required to delineate the mechanism(s) involved.

## References

[1] Shamsi M, Haghverdi F, Changizi- Ashtiyani S (2014). A brief review of Rhazes, Avicenna, and Jorjani’s views on diagnosis of diseases through urine examination. Iran J Kidney Dis.

[2] Fan PC, Chen CC, Chen YC, Chang YS, Chu PH (2016). MicroRNAs in acute kidney injury. Hum Genomics.

[3] Makris K, Spanou L (2016). Acute kidney injury: definition, pathophysiology and clinical phenotypes. Clin Biochem Rev.

[4] Mahmoudzadeh L, Najafi H, Changizi-Ashtiyani S, Mohamadi Yarijani Z (2017). Anti-inflammatory and protective effects of saffron extract in ischaemia/reperfusion-induced acute kidney injury. Nephrology.

[5] Ferenbach DA, Bonventre JV (2016). Acute kidney injury and chronic kidney disease: From the laboratory to the clinic. Nephrol Ther.

[6] Barnes CJ, Distaso CT, Spitz KM, Verdun VA, Haramati A (2016). Comparison of stem cell therapies for acute kidney injury. Am J Stem Cells.

[7] Bastani B (2015). The worsening transplant organ shortage in USA; desperate times demand innovative solutions. J Nephropathol.

[8] Huber-Lang M, Wiegner R, Lampl L, Brenner RE (2016). Mesenchymal stem cells after polytrauma: actor and target. Stem Cells Int.

[9] Aghajani Nargesi A, Lerman LO, Eirin A (2017). Mesenchymal stem cell-derived extracellular vesicles for kidney repair: current status and looming challenges. Stem Cell Res Ther.

[10] Shih YC, Lee PY, Cheng H, Tsai CH, Ma H, Tarng DC (2013). Adipose-derived stem cells exhibit anti-oxidative and antiapoptotic properties to rescue ischemic acute kidney injury in rats. Plast Reconstr Surg.

[11] Collett JA, Traktuev DO, Mehrotra P, Crone A, Merfeld-Clauss S, March KL (2017). Human adipose stromal cell therapy improves survival and reduces renal inflammation and capillary rarefaction in acute kidney injury. J Cell Mol Med.

[12] Herrera M, Mirotsou M (2014). Stem cells: Potential and challenges for kidney repair. Am J Physiol Renal Physiol.

[13] Hoseini SJ, Ghazavi H, Forouzanfar F, Mashkani B, Ghorbani A, Mahdipour E (2017). Fibroblast growth factor 1-transfected adipose-derived mesenchymal stem cells promote angiogenic proliferation. DNA Cell Biol.

[14] Tan J, Hu J, He Y, Cui F (2015). Protective role of silymarin in a mouse model of renal Ischemia-reperfusion injury. Diagn Pathol.

[15] Nemoto T, Burne MJ, Daniels F, O’Donnell MP, Crosson J, Berens K (2001). Small molecule selectin ligand inhibition improves outcome in ischemic acute renal failure. Kidney Int.

[16] Naccarato WF, Treuting JJ, Cannon DC (1981). Gravimetric determination of urine volumes. Am J Med Technol.

[17] Ashour RH, Saad MA, Sobh MA, Al-Husseiny F, Abouelkheir M, Awad A (2016). Comparative study of allogenic and xenogeneic mesenchymal stem cells on cisplatin-induced acute kidney injury in sprague-dawley rats. Stem Cell Res Ther.

[18] Azarkish F, Hashemi K, Talebi A, Kamalinejad M, Soltani N, Pouladian N (2017). Effect of the administration of solanum nigrum fruit on prevention of diabetic nephropathy in streptozotocin-induced diabetic rats. Pharmacognosy Res.

[19] Qasem MA, Noordin MI, Arya A, Alsalahi A, Jayash SN (2018). Evaluation of the glycemic effect of ceratonia siliqua pods (Carob) on a streptozotocin-nicotinamide induced diabetic rat model. Peer J.

[20] Najafi H, Changizi Ashtiyani S, Sayedzadeh SA, Mohamadi Yarijani Z, Fakhri S (2015). Therapeutic effects of curcumin on the functional disturbances and oxidative stress induced by renal ischemia/reperfusion in rats. Avicenna J Phytomed.

[21] Sheridan AM, Bonventre JV (2000). Cell biology and molecular mechanisms of injury in ischemic acute renal failure. Curr Opin Nephrol Hypertens.

[22] Sheridan AM, Bonventre JV (2001). Pathophysiology of ischemic acute renal failure. Contrib Nephrol.

[23] Chen YT, Sun CK, Lin YC, Chang LT, Chen YL, Tsai TH (2011). Adipose-derived mesenchymal stem cell protects kidneys against ischemia-reperfusion injury through suppressing oxidative stress and inflammatory reaction. J Transl Med.

[24] Abd Elwahab S, Hussein Ali A, Sayed Mahmoud A, Fathy Ahmed A, Foua d Ahmed R (2017). Bone marrow derived mesenchymalstem cell therapy in Induced acute renal injury in adult male albino rats. J Cytol Histol.

[25] Beiral HJ, Rodrigues-Ferreira C, Fernandes AM, Gonsalez SR, Mortari NC, Takiya CM (2014). The impact of stem cells on electron fluxes, proton translocation, and ATP synthesis in kidney mitochondria after ischemia/reperfusion. Cell Transplant.

[26] Monsel A, Zhu YG, Gennai S, Hao Q, Liu J, Lee JW (2014). Cell-based therapy for acute organ injury: preclinical evidence and ongoing clinical trials using mesenchymal stem cells. Anesthesiology.

[27] Shen ZY, Zhang J, Song HL, Zheng WP (2013). Bone-marrow mesenchymal stem cells reduce rat intestinal ischemia-reperfusion injury, ZO-1 downregulation and tight junction disruption via a TNF-alpha-regulated mechanism. World J Gastroenterol.

[28] Changizi Ashtiyani S, Zohrabi M, Hassanpoor A, Hosseini N, Hajihashemi S (2013). Oral administration of the aqueous extract of rosmarinus officinalis in rats before renal reperfusion injury. Iran J Kidney Dis.

[29] Moosavi SM, Bayat G, Owji SM, Panjehshahin MR (2009). Early renal post-ischaemic tissue damage and dysfunction with contribution of A1-adenosine receptor activation in rat. Nephrology (Carlton).

[30] Seguro AC, Shimizu MH, Monteiro JL, Rocha AS (1989). Effect of potassium depletion on ischemic renal failure. Nephron.

[31] La Manna G, Bianchi F, Cappuccilli M, Cenacchi G, Tarantino L, Pasquinelli G (2011). Mesenchymal stem cells in renal function recovery after acute kidney injury: use of a differentiating agent in a rat model. Cell Transplant.

[32] Nakamura K, Komagiri Y, Kubokawa M (2012). Effects of cytokines on potassium channels in renal tubular epithelia. Clin Exp Nephrol.

[33] Rowart P, Erpicum P, Detry O, Weekers L, Gregoire C, Lechanteur C (2015). Mesenchymal stromal cell therapy in ischemia/reperfusion injury. J Immunol Res.

[34] Yang CC, Chien CT, Wu MH, Ma MC, Chen CF (2008). NMDA receptor blocker ameliorates ischemia-reperfusion-induced renal dysfunction in rat kidneys. Am J Physiol Renal Physiol.

[35] Fonseca I, Reguengo H, Almeida M, Dias L, Martins LS, Pedroso S (2014). Oxidative stress in kidney transplantation: malondialdehyde is an early predictive marker of graft dysfunction. Transplantation.

[36] Moosavi SMS, Changizi-Ashtiyani S, Hosseinkhani S (2011). L-carnitine improves oxidative stress and suppressed energy metabolism but not renal dysfunction following release of acute unilateral ureteral obstruction in rat. Neurol Urodyn.

[37] Zhuo W, Liao L, Xu T, Wu W, Yang S, Tan J (2011). Mesenchymal stem cells ameliorate ischemia-reperfusion-induced renal dysfunction by improving the anti-oxidant/oxidant balance in the ischemic kidney. Urol Int.

[38] Zhang Z, Dmitrieva NI, Park JH, Levine RL, Burg MB (2004). High urea and NaCl carbonylate proteins in renal cells in culture and in vivo, and high urea causes 8-oxoguanine lesions in their DNA. Proc Natl Acad Sci U S A.

[39] Mohammadzadeh M, Halabian R, Gharehbaghian A, Amirizadeh N, Jahanian-Najafabadi A, Roushandeh AM (2012). Nrf-2 overexpression in mesenchymal stem cells reduces oxidative stress-induced apoptosis and cytotoxicity. Cell Stress Chaperones.

[40] Kim WS, Park BS, Kim HK, Park JS, Kim KJ, Choi JS (2008). Evidence supporting anti-oxidant action of adipose-derived stem cells: protection of human dermal fibroblasts from oxidative stress. J Dermatol Sci.

[41] Havasi A, Borkan SC (2011). Apoptosis and acute kidney injury. Kidney Int.

[42] Gnecchi M, Zhang Z, Ni A, Dzau VJ (2008). Paracrine mechanisms in adult stem cell signaling and therapy. Circ Res.

[43] Glenn JD, Whartenby KA (2014). Mesenchymal stem cells: Emerging mechanisms of immunomodulation and therapy. World J Stem Cells.

[44] Lange C, Togel F, Ittrich H, Clayton F, Nolte-Ernsting C, Zander AR (2005). Administered mesenchymal stem cells enhance recovery from ischemia/reperfusion-induced acute renal failure in rats. Kidney Int.

[45] Patschan D, Buschmann I, Ritter O, Kribben A (2018). Cell-Based therapies in acute kidney injury (AKI). Kidney Blood Press Res.

[46] Bonventre JV, Yang L (2011). Cellular pathophysiology of ischemic acute kidney injury. J Clin Invest.

[47] Kelly KJ, Williams WW, Colvin RB, Meehan SM, Springer TA, Gutierrez-Ramos JC (1996). Intercellular adhesion molecule-1-deficient mice are protected against ischemic renal injury. J Clin Invest.

[48] Hara Y, Stolk M, Ringe J, Dehne T, Ladhoff J, Kotsch K (2011). In vivo effect of bone marrow-derived mesenchymal stem cells in a rat kidney transplantation model with prolonged cold ischemia. Transpl Int.

[49] Goligorsky MS, Brodsky SV, Noiri E (2002). Nitric oxide in acute renal failure: NOS versus NOS. Kidney Int.

[50] Peresleni T, Noiri E, Bahou WF, Goligorsky MS (1996). Antisense oligodeoxynucleotides to inducible NO synthase rescue epithelial cells from oxidative stress injury. Am J Physiol.

[51] Hagiwara M, Shen B, Chao L, Chao J (2008). Kallikrein-modified mesenchymal stem cell implantation provides enhanced protection against acute ischemic kidney injury by inhibiting apoptosis and inflammation. Hum Gene Ther.

[52] Borger V, Bremer M, Ferrer-Tur R, Gockeln L, Stambouli O, Becic A (2017). Mesenchymal stem/stromal cell-derived extracellular vesicles and their potential as novel immunomodulatory therapeutic agents. Int J Mol Sci.

[53] Song Y, Peng C, Lv S, Cheng J, Liu S, Wen Q (2017). Adipose-derived stem cells ameliorate renal interstitial fibrosis through inhibition of EMT and inflammatory response via TGF-β1 signaling pathway. Int Immunopharmacol.

[54] Eirin A, Zhu XY, Krier JD, Tang H, Jordan KL, Grande JP (2012). Adipose tissue-derived mesenchymal stem cells improve revascularization outcomes to restore renal function in swine atherosclerotic renal artery stenosis. Stem Cells.

[55] Sheashaa H, Lotfy A, Elhusseini F, Aziz AA, Baiomy A, Awad S (2016). Protective effect of adipose-derived mesenchymal stem cells against acute kidney injury induced by ischemia-reperfusion in Sprague-Dawley rats. Exp Ther Med.

